# Undergraduate Medical Students Using Facebook as a Peer-Mentoring Platform: A Mixed-Methods Study

**DOI:** 10.2196/mededu.5063

**Published:** 2015-10-27

**Authors:** Severin Pinilla, Leo Nicolai, Maximilian Gradel, Tanja Pander, Martin R Fischer, Philip von der Borch, Konstantinos Dimitriadis

**Affiliations:** ^1^Institut für Didaktik und Ausbildungsforschung in der MedizinKlinikum der Universität MünchenLudwig-Maximilians-Universität MünchenMunichGermany

**Keywords:** medical education, peer mentoring, social media, Facebook

## Abstract

**Background:**

Peer mentoring is a powerful pedagogical approach for supporting undergraduate medical students in their learning environment. However, it remains unclear what exactly peer mentoring is and whether and how undergraduate medical students use social media for peer-mentoring activities.

**Objective:**

We aimed at describing and exploring the Facebook use of undergraduate medical students during their first 2 years at a German medical school. The data should help medical educators to effectively integrate social media in formal mentoring programs for medical students.

**Methods:**

We developed a coding scheme for peer mentoring and conducted a mixed-methods study in order to explore Facebook groups of undergraduate medical students from a peer-mentoring perspective.

**Results:**

All major peer-mentoring categories were identified in Facebook groups of medical students. The relevance of these Facebook groups was confirmed through triangulation with focus groups and descriptive statistics. Medical students made extensive use of Facebook and wrote a total of 11,853 posts and comments in the respective Facebook groups (n=2362 total group members). Posting peaks were identified at the beginning of semesters and before exam periods, reflecting the formal curriculum milestones.

**Conclusions:**

Peer mentoring is present in Facebook groups formed by undergraduate medical students who extensively use these groups to seek advice from peers on study-related issues and, in particular, exam preparation. These groups also seem to be effective in supporting responsive and large-scale peer-mentoring structures; formal mentoring programs might benefit from integrating social media into their activity portfolio.

## Introduction

Peer mentoring is a well-established core element for creating supportive learning environments and facilitating successful careers in medicine at different stages of medical training [1-3]. Studies in other areas of higher education have shown that students might have a particular need for peer mentoring during the first time of transition to another academic institution in order to adapt to the new learning and teaching environment and the specific struggles they face [4,5]. It has also been acknowledged that peer mentoring is essential for a satisfying medical school experience by junior students in particular [6,7].

There are promising data on how medical students who are involved in peer teaching benefit from these programs [8-11]. However, the difference between peer teaching and peer mentoring remains unclear in the context of medical students, and aspects such as mutual emotional support and empowerment usually are not addressed in these studies.

At the same time, there is an important change in terms of how undergraduate students in different disciplines use and take advantage of virtual platforms and networks such as Facebook within formal and informal mentoring contexts [12-16]. Studies in other contexts of higher education indicate that social media might play an important role for becoming part of a community in a new environment and forming educational microcommunities [17-19]. Educators are increasingly becoming aware of the potential of integrating social media into formal teaching and learning environments for a diverse range of activities [20-23].

However, there is a paucity of data that provide information on whether and how undergraduate medical students use Facebook for peer mentoring in a way that goes beyond peer teaching. It also remains unclear whether Facebook as a potential peer mentoring tool merely serves as an information-sharing platform or whether its affordances actually provide an effective peer-mentoring environment and might support, counteract, or replace institutional learning-management systems and face-to-face peer mentoring.

The purpose of this mixed-methods study is to provide a definition of peer mentoring in the context of undergraduate medical education as well as to quantify and explore the use and perspectives of medical students with regard to Facebook as a virtual peer-mentoring environment. The data might help medical educators to leverage the potential and spotlight limitations of social media for peer-mentoring programs and provide guidance for formulating best practice recommendations for integrating social media into formal peer-mentoring programs.

## Methods

### Overview

A mixed-methods approach was chosen to provide a multidimensional representation of peer-mentoring activities of medical students on Facebook [24]. An expert group consisting of medical educators, clinicians, and undergraduate and graduate medical students discussed and reviewed the literature [11,25-28] to develop the following peer-mentoring definition and derive coding anchors.

Peer mentoring is a form of mentoring that involves informal dynamic relationships within a group of individuals who are similar in experience and rank. It is based on the premise that there is a pool of skills, experiences, and resources within the group that is deliberately or subliminally used to support and empower one another and to foster everyone's development. Because of the equality among group members, relationships are generally personal and mutual, and ideally, each participant has something of value to contribute and gain.

The personal experience of the authors with using Facebook groups and word-of-mouth advice from currently enrolled preclinical students was used to identify the relevant Facebook groups; these were initiated and used by undergraduate medical students at Ludwig-Maximilians-Universität (LMU) Munich, Germany, in the first (PCY1) and second preclinical year (PCY2). Several small Facebook groups and two large groups were found. The authors identified one Facebook group per preclinical year as the main group, based on structure and number of members as compared to the currently enrolled student cohorts. All posts and comments in both groups of a complete academic semester—from September 2013 through February 2014—were included in the analysis. The two identified member groups in Facebook were both initiated by peers of undergraduate students, who commenced their medical studies in October 2013 (referred to as preclinical year 1, PCY1) and in October 2012 (referred to as preclinical year 2, PCY2). Both groups had self-identifying names and new members needed to apply and then be accepted by persons already in the group. All posts and comments in these groups were extracted and exported into Microsoft Excel. We then conducted a quantitative analysis of submissions per week and month.

A social constructivist perspective was applied to identify emerging peer-mentoring themes that are relevant for undergraduate medical students and reflect social norms, values, and needs of medical students in this context. Based on the working definition of peer mentoring, the authors additionally developed a coding scheme for content analysis of posts and comments (see Table 1) [29]. Anchoring examples were defined for each peer-mentoring subcategory and the final coding scheme was applied to three critical weeks of each preclinical year with particularly high or low posting frequency, as well as at the time of high-stakes exams. The research team agreed on analyzing data from the beginning of each semester, time periods directly correlating to critical written and oral exams, and to contrast these weeks with posting-behavior between examination periods.

**Table 1:** Peer-mentoring coding scheme^a^.

Categories and subcategories		Anchoring example
**Study related**		
	Knowledge/skills	“In the beginning you should focus on learning the musculoskeletal structures instead of worrying about electives”
	Experiences	“I have done my first preclinical rotation in the university hospital on a neurology ward and was amazed by the attendings’ willingness to teach”
	Resources	“You can find a selection of preparatory exams in the anatomy exam folder on the online-learning platform Moodle”
	Emotional support	“It’s absolutely normal to be afraid of the terminology exam and the Latin grammar questions, but it is really easier than you think”
**Nonstudy related**		
	Social activities	“Let’s meet at my place before going to the freshmen party”
	Advertising	“Medical education books at a special discount for new students”

^a^All posts were additionally coded in the categories “Exams and learning,” “Study logistics and organization,” and “Extracurricular activities.”

Quantitative analysis of posting patterns in Facebook groups was used to describe the posting behavior across academic semesters. Thematic content analysis of Facebook posts and focus group transcripts was used to explore posting content and perceptions of undergraduate medical students with regard to peer-mentoring categories (see Table 1). Some posts and comments included more than one peer-mentoring theme and consequently were coded for all identifiable subcategories. After familiarization with the full qualitative and quantitative dataset, data were contrasted and used to enhance and strengthen the final interpretation, as well as to inform the discussions on emerging differences with regard to interpretation of data and applying the coding scheme. All differences were discussed and resolved.

Participants for focus groups were recruited through email invitations at LMU (total n=21) and two focus groups with medical students enrolled in different semesters were conducted. Both focus group discussions were based on a written protocol, facilitated by an experienced researcher, recorded, and documented. The recordings of both focus groups were fully transcribed and coded. The selected dataset was coded twice in an independent manner. Interrater reliability and Cohen’s kappa were calculated [30] using Stata software version 12 (StataCorp LP).

### Ethics

The LMU ethics committee reviewed the research design and exempted the study from additional ethical approval. Confidentiality and anonymity with regard to electronic data was maintained throughout the study. Any names or potentially identifying information were removed before analyzing the data and quotes were all translated from German to English for this manuscript. Pseudonyms were used to maintain confidentiality and anonymity.

## Results

### Characteristics of Analyzed Facebook Groups

At the time of collecting the data, the PCY1 Facebook group had 1149 members, of which 728 (63.36%) were active users who contributed at least one post throughout the semester. The PCY2 group had a total of 1213 members, of which 863 (71.15%) were active users who contributed at least one post throughout the semester. The combined groups consisted of 2362 counted group members, with several individuals being members in both groups, however. The corresponding student cohorts enrolled at the LMU Munich in the respective preclinical years consisted of 561 female and 389 male students in PCY1 (total of 950), and 569 female and 397 male students in PCY2 (total of 966). The full sample of enrolled undergraduate medical students therefore consisted of 1916 individuals.

### Posting Activity of Preclinical Medical Students

A total of 5939 posts, of which 1168 (19.67%) represented primary posts, were extracted from the PCY1 Facebook group. The total number of posts (n=11,853) is the sum of primary posts and all reply posts and comments. Out of all posted questions in the PCY1 group, 79.5% (116/146) were answered in a satisfactory way by peer students. Similarly, a total of 5914 posts with 1246 (21.07%) primary posts were extracted from the PCY2 Facebook group. A total of 76.8% (116/151) of all posted questions in the PCY2 group were answered in a satisfactory way. With regard to posting frequency of group members, 79.70% (1268/1591) of active users in both groups posted 1-10 posts during the extracted period, accounting for 36.11% (4280/11,853) of all posts. A total of 16.40% (261/1591) of active users accounted for another 38.56% (4570/11,853) of posts (11-30 posts) and, finally, a minority of 3.90% (62/1591) of active users accounted for the remaining 25.34% (3003/11,853) of posts (>30 posts).

**Figure 1:**
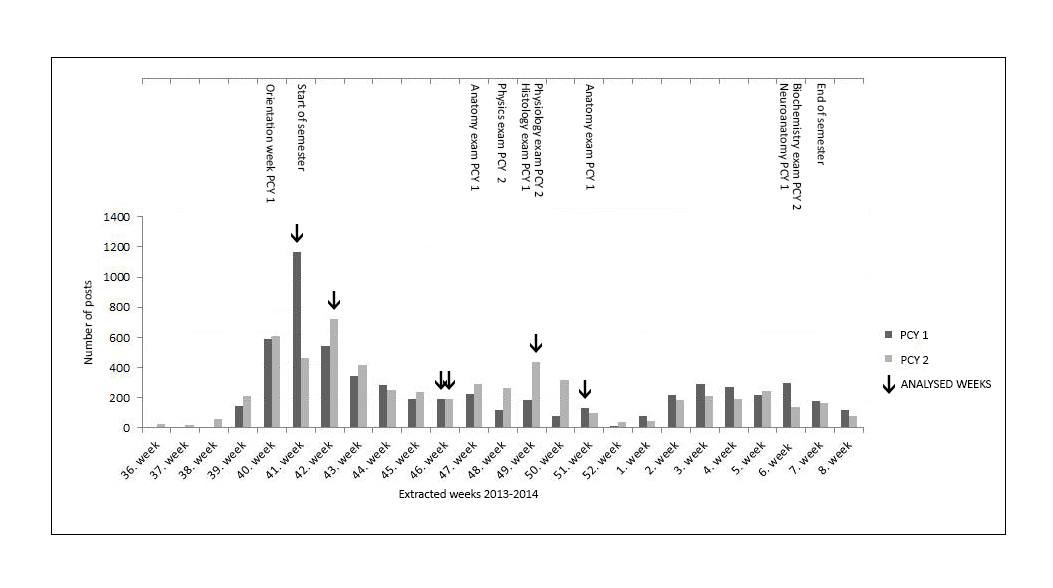
Facebook posting pattern by undergraduate medical students.

All posts were charted over time and relevant curriculum elements—orientation week, exams, and duration of semester—were identified (Figure 1). Based on the posting activity during the semesters, 3 relevant exemplary weeks in each group were identified (marked in Figure 1) and used for detailed thematic content analysis: one week showed a particularly high posting activity (the beginning of each semester), a second week showed low posting activity (not correlating with any critical exam), and a third week was directly correlated to an exam period. In addition, we found that students in both Facebook groups started posting 2 weeks (PCY1) and 5 weeks (PCY2) before the official beginning of the semesters, respectively.

### Peer-Mentoring Elements in Facebook Groups

Our final peer-mentoring coding scheme showed good reliability—average Cohen’s kappa of .77 (SD .16) and 94% agreement across all main categories, and Cohen's kappa of .62 (SD .27) and 82% agreement for all subcategories. Peer-mentoring elements of all categories were identified in both Facebook groups. The dominant themes in both preclinical years were knowledge- and skills-related posts and comments, as well as resources-related posts (see Table 2).

**Table 2:** Frequency of peer-mentoring elements in posts and comments.

Categories and subcategories	PCY1^a^, % average frequency^b^ (SD)	PCY2^c^, % average frequency^b^ (SD)
Study related	76 (5)	75 (4)
Knowledge/skills	10 (4)	65 (3)
Experiences	14 (15)	1 (1)
Resources	68 (18)	33 (3)
Emotional support	8 (7)	1 (2)
Nonstudy related	24 (5)	25 (4)
Social activities	99 (2)	84 (3)
Advertising	1 (2)	16 (3)

^a^First preclinical year (PCY1).

^b^Percentages represent the averages across all analyzed weeks in each preclinical year.

^c^Second preclinical year (PCY2).

The following excerpts (translated from German) illustrate how students discuss educational and coursework aspects, which are relevant for choosing their anatomy books, and what strategies work in order to prepare for the anatomy exams:

Alright...so meanwhile, I can go and get the lecture notes...and I just walk in there and say, “I would like to have the lecture notes for anatomy?” :D Would you also recommend that I buy or borrow an atlas of anatomy?

Student F, PCY1

Yes that’s how you do it :D Borrowing never hurts, I would say.

Student W, PCY2

Alright, thanks :)

Student F, PCY1

I would definitely recommend [brand name]! Although it’s a bit more expensive, it is way better from a didactic perspective, and it has better illustrations, which are really helpful =)...you might not even need any other textbook if you really work with it...and the different levels are illustrated in a way so that they match, like the images of muscles exactly fit the images of bones and so forth, and that really helps to get the picture.

Student C, PCY2

All other nonstudy-related posts—24% (SD 5) and 25% (SD 4)in PCY1 and PCY2, respectively—were coded as social activities, such as extracurricular activities or any other type of social events:

Hey everyone, so I think I missed some things. I just found...that there is a newbies party tonight at [bar’s name], anything else that I have missed?

Student D, PCY1

With regard to posting patterns, we observed a shift of both frequency and content focus in both preclinical years. In PCY1, undergraduate students started off posting questions predominantly related to learning strategies; later during the semester they changed the focus of posts to questions on written and oral exams—72.4% (105/145) versus 19% (6/32) of all posts on learning strategies, and 11.0% (16/145) versus 56% (18/32) of all posts on exams, comparing early and late weeks of semesters, respectively:

Ahhh! I don’t get the physics lecture notes! :’( Are the problems’ solutions available somewhere within the notes? Or a self-help/study group, with a free spot?

Student E, early post in PCY2

You have to...be able to do the math in front of the class, if those problems are part of your oral test. But here's some advice: You can find physics tutorials online on Moodle [learning management system] and you can also find most of the solutions there as well ;)

Student G, response to student E, PCY2

Hey, another question—do striated muscle cells have a calcium-dependent plateau phase? Thanks guys, without you it would be quite tough here :)

Student Y, late post in PCY2

Nope, only the heart muscle cells as far as I know

Student Q, response to student Y, PCY2

NO! That is typical for heart muscle cells!!!

Student P, response to student Y, PCY2

Puh, thanks—that is a huge piece of the puzzle that I was missing ;)

Student Y, response to students Q and P, PCY2

### Peer-Mentoring Elements in Medical Students’ Perspectives of Facebook

The thematic content analysis of the focus group discussions confirmed our findings from direct Facebook group analysis and exploration. All peer-mentoring categories emerged in the focus group discussions.

#### Theme 1: Similarity in Experience and Rank

Participants confirmed the high intensity of use of Facebook groups among peers within one semester in particular during the time of exams:

Well, you know, before exams, I really liked that people discussed the questions from previous exams, and that people tried to find the right answers, that was really helpful when preparing for exams.

Person 6

I really found it cool, because there were always people who made an effort, and provided awesome summaries for exams and so forth.

Person 8

In addition, students developed a strategy to form course-specific groups for each academic course during a semester in order to make the content more specific to their needs:

You know...it really has advantages...like in Module 23 or Module 4 [refers to courses in different semesters], there are so many subjects and fast changes. If that would be one big Facebook group, it would be pure chaos.

Person 3

Students also agreed that group members were more or less similar in rank and experience, which in their opinion was reflected in intense contextual discussions:

Yeah, it also happens...like when two students...that is when one thinks he is right and the other one thinks it’s wrong, then they really get a discussion going...like with 30 comments or so...but usually it’s only a few posts until the right answer is online.

Person 4

#### Theme 2: Pool of Skills, Experiences, and Resources Within the Group

One participant outlined the importance of Facebook membership in particular when students missed lectures or did not like to go to lectures in general, and relied on the relevant information and resources being shared within the Facebook groups:

You know...those students who never go to lectures definitely benefit from other students who share their knowledge [in Facebook groups] from having gone to the lectures.

Person 2

In addition, participants seemed to perceive Facebook predominantly as a tool to navigate their studies and to manage access to relevant information:

Well, I am basically only in study Facebook groups...so that you get all the information for the clinical rotations or for the exams, and so that you don’t miss deadlines, because there are always some students who nicely post everything, so that you don’t miss anything...that’s pretty nice.

Person 1

Some comments from participants indicated that Facebook groups are an important source to learn about the hidden curriculum and have an impact on the learning strategy and behavior of students:

You know...like the unofficial information, like...do they really check whether you are present or not, or is it rather a seminar, where people don’t realize that you are absent, I mean...it’s pretty easy to get that kind of information through Facebook groups and you wouldn’t know that otherwise.

Person 5

Yeah right, and students who have done the course already...you know, they share tips and tricks...which is particularly helpful when preparing for oral exams like in anatomy...like people posting lists with the main questions that are being asked, you know.

Person 10

Comments on feeling mentored in the Facebook groups referenced the peer group sources as "swarm intelligence" and feeling "swarm mentored" as a consequence of it:

You know, in pharmacology...there were one or two brains [refers to very good students] and they went back and forth...even with literature searches, only so that they could answer the question [refers to pharmacology questions posted in a Facebook group] it was like...I would say swarm intelligence...and that’s [refers to feeling mentored] sort of swarm mentoring...just like swarm intelligence.

Person 4

#### Theme 3: Empowerment and Emotional Support

Empowerment and emotional support as elements of peer mentoring were present as well in the perceptions of participants:

[Talking about advantages of being a member of the Facebook groups] You know, sort of team work, like people are not working against each other but with each other. So that you can find the solutions or exchange thoughts and helping, you know

Person 1

One participant described the Facebook groups as a "system" of support:

Well, I sort of feel taken care of, like when I have the feeling that there is a system, that helps me become more self-confident, so that I can understand how things work and I am being prepared [refers to exams].

Person 10

However, the exposure of personal information and making a fool of oneself in front of a larger group were partly perceived as difficult in the context of Facebook groups:

It’s also kind of a data safety question. Who really wants personal things to be in the Internet forever?

Person 10

You know, and maybe...you actually know each other, and you don’t want to show yourself up, sort of.

Person 2

At the same time, there seemed to be some sort of self-regulation present within the groups as well:

Usually, those posts that are like bad low blows...those are usually removed quickly, although you find those, too.

Person 10

## Discussion

### Principal Findings

Our data indicate that all peer-mentoring elements as defined above and that are based in the literature on mentoring in medical education [11,25-28] are present in Facebook groups that are formed by preclinical undergraduate medical students. The fact that many, if not most, preclinical medical students are already members of Facebook and can make use of its multiple affordances, such as forming closed groups and exchanging diverse online media, makes Facebook an interesting supportive platform for peer-mentoring programs. The content of these informal groups, generally formed by students with similar rank and experience, seems to be beneficial for all members of the group. A pool of skills, experiences, and resources is built through these groups in order to facilitate access for all members. The posting pattern within these groups reflects the curriculum design, and exams seem to be a trigger for increasing posting intensity.

The differences in Facebook group sizes most likely result from some students not leaving the Facebook groups after completing the semesters. However, students constantly seem to adjust the form of these groups according to their educational needs. Further studies are needed to better characterize the life cycle of these groups.

A rather small group consisting of 3.90% (62/1591) of students was accountable for about 25.34% (3003/11,853) of all posts, indicating that some students might function as social media drivers. The vast majority (1268/1591, 79.70%) of students showed less active posting behavior, still membership was identified as essential during focus group discussions in order to have access to all relevant information. The vast majority of all questions, that is 78.1% (232/297), posted in both groups (PCY1 and PCY2) were answered satisfactorily and demonstrate a responsive and functional network.

However, more complex peer-mentoring elements such as empowering and fostering personal development seem to be underrepresented in these groups. Further research is needed to better understand the specific role of formal peer-mentoring programs in this context and how formal peer-mentoring programs should be combined with online social networks.

Social network sites like Facebook might provide useful affordances for peer-mentoring programs, in particular for large medical faculties. Based on the observation that Facebook groups of undergraduate medical students are used more in order to share resources and experiences as compared to emotional support, we assume that real-life social networks play an at least equally important role for peer-mentoring activities. Furthermore, our data showed that students start forming these groups even before the official start of semesters. Medical faculties might thus also use these groups for communicating relevant orientation information to preclinical students, although official involvement of faculty might alter the acceptance and dynamics of these groups.

Specifically trained social media educators might thus help to support a positive learning culture and guide students more effectively toward relevant resources and faculty members, since informal Facebook groups rather lack structure, organization, and quality control of information. Finally, aspects like data privacy and digital professionalism need to be considered carefully [31]. Further research is needed to better understand involvement and perceptions of medical students with regard to Facebook and to what extent formal involvement of faculty is needed and accepted as compared to other forms of peer mentoring [15].

### Conclusions

Our findings indicate an extensive use and, so far, not well-understood role of social network sites in the context of peer mentoring for undergraduate medical students. The fact that many, if not most, preclinical medical students are already members of Facebook and can make use of its multiple affordances, such as forming closed groups and exchanging diverse online media, makes Facebook an interesting supportive platform for peer-mentoring programs.

Although Facebook might not be able to replace all face-to-face elements of peer-mentoring programs, we found that medical students’ extensive informal use of Facebook could be leveraged to support formal peer-mentoring programs for undergraduate medical students. The typology and behavior of subgroups within the identified Facebook groups are not fully understood and the adequate roles of faculty members and official mentoring programs in the context of social media require further exploration.

## Abbreviations

LMU: Ludwig-Maximilians-Universität
PCY1: first preclinical year
PCY2: second preclinical year
